# Measuring radiation-induced DNA damage in *Cryptococcus neoformans* and *Saccharomyces cerevisiae* using long range quantitative PCR

**DOI:** 10.1371/journal.pone.0207071

**Published:** 2018-11-08

**Authors:** Wanchang Cui, XiangHong Li, Lisa Hull, Mang Xiao

**Affiliations:** Radiation Countermeasures Program, Armed Forces Radiobiology Research Institute, Uniformed Services University of the Health Sciences, Bethesda, MD, United States of America; University of Michigan Health System, UNITED STATES

## Abstract

DNA damage has been considered to be the universal critical lesion in cells after exposure to ionizing radiation. Measuring radiation-induced DNA damage is important to understand the mechanisms of radiation-induced toxicity and monitor DNA damage repairs. Currently the most widely used methods to measure DNA damage are pulsed-field gel electrophoresis (PFGF) and single-cell gel electrophoresis (also known as the comet assay), both of which are technically challenging and time consuming. Long range quantitative polymerase chain reaction (LR-QPCR) has been used successfully to measure nuclear and mitochondrial DNA damage in mammalian and several model organism cells. The principle of this assay is that DNA lesions will slow down or block the progression of DNA polymerase. Therefore, the amplification efficiency of DNA with fewer lesions will be higher than DNA with more lesions under the same reaction condition. Here, we developed the LR-QPCR assay primers and reaction conditions to quantify DNA damage in *Cryptococcus neoformans* (*C*. *neoformans*) and *Saccharomyces cerevisiae* (*S*. *cerevisiae*) after gamma ray exposure. Under these conditions, long DNA targets of *C*. *neoformans* H99 and *S*. *cerevisiae* BY4741 (17.6 and 16.4 kb for nuclear DNA and 15.3 and 14.6 kb for mitochondrial DNA) were quantitatively amplified using extracted DNA templates, respectively. Two short mitochondrial DNA targets of these two species (207 bp and 154 bp) were also quantitatively amplified and used to monitor the number of mitochondria. Using the LR-QPCR method, we showed that the frequency of radiation-induced mitochondrial and nuclear DNA lesions had a significant linear correlation with the radiation doses (from 500 Gy to 3000 Gy) in both species. Furthermore, the faster disappearance of DNA damage detected in *C*. *neoformans* H99S strain compared to H99 strain may help to explain the different radiation sensitivity of these two strains. In summary, we developed a simple, sensitive method to measure radiation-induced DNA damage, which can greatly facilitate the study of radiation-induced toxicity and can be widely used as a dosimetry in radiation-induced cell damage.

## Introduction

It has been well established that DNA damage is the universal critical lesion in cells after exposure to ionizing radiation [1, 2]. Ionizing radiation can directly cause DNA damage by depositing energy of photons, or indirectly cause DNA damage by generating free radicals. The major types of DNA damage induced by ionizing radiation include double strand breaks (DSB), single strand breaks (SSB), and base damages. Ionizing radiation causes quantitative DNA damage. It has been estimated that ionizing radiation induces around 850 pyrimidine lesions, 450 purine lesions, 1000 SSB and 20–40 DSB/cell/Gy with low linear energy transfer (LET) γ-radiation in mammalian cells [3]. It was also reported that ionizing radiation generates about 0.004 DSB/Mbp/Gy across broadly phylogenetically diverse cell-types (including plasmid, virus, mammalian cells and the most radiation resistant organisms) [4]. Pulsed-field gel electrophoresis (PFGF) and single-cell gel electrophoresis (also known as the comet assay) are the two most widely used methods to measure DNA damage. However, both methods are technically-challenging and time consuming [5, 6].

The polymerase chain reaction (PCR) method has been tried in the 1990s to detect DNA damage based on the idea that DNA damage would block DNA polymerase progression [7, 8]; however, because of the technical limitation at that time, only very short DNA fragments were amplified to detect DNA damage (<500 bp and 2 kb). Radiation-induced DNA damage is generally a rare event. For example, ionizing radiation causes about 3000 DNA damages/mammalian cell/Gy [9], which equals to about 1 DNA damage/Mbp/Gy, therefore a long DNA fragment needs to be amplified in order to catch the DNA damage. Recently, new DNA polymerases suitable for long DNA fragment amplification have been developed and long range quantitative PCR (LR-QPCR) has been used successfully to measure DNA damage in virus, mammalian and several model organism cells [10–12]. The principle of this assay is that DNA lesions will slow down or block the progression of DNA polymerase. Therefore, the amplification efficiency of DNA with fewer lesions will be higher than DNA with more lesions, and very low proportion of amplification may occur in severe DNA damaged samples under the same reaction condition. The main challenge for the LR-QPCR method is the need to amplify long range DNA fragment (generally > 10 kb) so that the potential DNA damage can be detected. Recently, a wide range of commercially available long-range DNA polymerase is becoming available [13], therefore the LR-QPCR method can be performed in a general molecular laboratory.

The fungus *C*. *neoformans* is highly radiation resistant and has been found in highly radioactive environments such as the cooling pools of nuclear reactors, the stratosphere, and the damaged nuclear reactor at Chernobyl Nuclear Power Plant [14]. The *C*. *neoformans* var. *grubii* (H99) strain is the most tolerant to γ-radiation with a D_10_ value about 2000 Gy (D_10_ is the radiation dose yielding 10% survival) compared to other pathogenic *Cryptococcus* species and ascomycete nonpathogenic and pathogenic yeasts [15]. Many independent lineages of H99 were developed in different laboratories after the original isolation of H99 [16]. A higher-virulent variant, H99S, was derived via passages of a mixed H99 frozen stock through the well-validated rabbit model of central nervous system infection [16]. In comparison with the H99 fungus, *S*. *cerevisiae* is a less radioresistant yeast species. *S*. *cerevisiae* BY4741 (BY4741), a commonly used laboratory strain of *S*. *cerevisiae* was tolerant to γ-radiation with a D_10_ value about 1000 Gy [17].

In the current study, we developed the LR-QPCR method to detect and quantitate radiation-induced nuclear DNA and mitochondrial DNA damage in *C*. *neoformans* H99 and *S*. *cerevisiae* BY4741, and used this method to study DNA damage in these two species exposed to different doses of ionizing radiation. We further compared the DNA damage progression in H99S and H99 cells after radiation exposure, which may help explain their different radiation sensitivity.

## Materials and methods

### Strain and media

*C*. *neoformans* var. *grubii* H99 (ATCC 20882) was purchased from American Type Culture Collection (ATCC, Manassas, Virginia). *C*. *neoformans* H99S was a gift from Dr. Joe Heitman’s lab at Duke University Medical Center (Durham, North Carolina). These strains were routinely grown at 30°C in yeast extract peptone dextrose (YPD) medium. *S*. *cerevisiae* BY4741 was a gift from Dr. Michael Daly’s lab at Uniformed Services University of the Health Sciences (Bethesda, Maryland) and was routinely grown at 30°C in YPD medium [17].

### Irradiation with γ-rays

For molecular biology assays, overnight culture of fungal cells were washed in PBS and then irradiated in tubes on ice in a ^60^Co irradiator (Model 109; J. L. Shepard and Associates, San Fernando, California) at 130 Gy/min. The irradiated cells were either harvested immediately or diluted in 50 x volume of fresh YPD medium and harvested at selected time points after incubation with shaking.

For survival assays, overnight culture of fungal cells were 10-fold serially diluted in PBS and 3 μL of 10^2^−10^5^ dilutions were spotted onto YPD plates. The plates were irradiated in the same irradiator and the cells were allowed to grow for 5 days before being photographed.

### DNA extraction

DNA isolation from *C*. *neoformans* is difficult due to their thick and resistant capsule. *C*. *neoformans*’ DNA was isolated from the fungal cells using an urea-chelex method described by Gonzales et al. [18] and Mseddi et al [19]. Briefly, the cells were recovered by centrifugation from 15 mL of YPD culture shaking at 200 rpm overnight, washed once with cold water, and then incubated 3 h in 2 mL of urea buffer (urea 8 M, NaCl 0.5M, Tris 20 mM, EDTA 20 mM, SDS 2%, pH 8) at room temperature under agitation. Cells were then centrifuged 2 min at 4000 x g. The pellet was re-suspended in 300 μL of distilled water in a microcentrifuge tube. A volume of 100 μL of Chelex solution (10% Chelex-100 [Bio-Rad, Hercules, California] in an aqueous solution of 0.1% SDS, 1% Nonidet P-40, and 1% Tween 80) was added. The tubes were incubated at 95°C for 30 min and then on ice for 5 min. DNA was in the supernatant after 5 min of centrifugation (10,000 rpm) and stored at -20°C before use.

For *S*. *cerevisiae* BY4741 cells, DNA extraction was performed using the K0512 Genomic DNA Purification Kit (Thermo Fisher Scientific, Grand Island, New York) in this study. Briefly, the yeast cells were recovered by centrifugation from 15 mL of YPD culture shaking at 200 rpm overnight, and then suspended in 200 μL of TE buffer. The cells were lysed in 400 μL of lysis solution at 65^°^C for 5 min and then emulsified with 600 μL of chloroform. After spin down, the upper aqueous phase containing DNA was transferred to a new tube and 800 μL of freshly prepared precipitation solution was added. The tubes were centrifuged again and the supernatant was removed completely. The DNA pellet was dissolved in 100 μL NaCl solution and then precipitated with addition of 300 μL of cold ethanol and then kept at -20^°^C for at least 10 min. After spin down, the DNA pellet was washed again in 70% ethanol. Finally, the DNA was dissolved in 100 μL TE buffer and stored at -20°C before use.

### DNA quantification

DNA sample concentrations were determined by fluorescence measurements after DNA staining with PicoGreen (Quant-iT PicoGreen dsDNA reagent and kit; Thermo Fisher Scientific, New York, NY). Briefly, DNA samples were diluted in TE buffer according to the manufacturer’s manual and 100 μL aliquots were pipetted into microplate wells. The working PicoGreen solution was prepared by 1:200 dilution of the stock with TE buffer. Equal amounts (100 μL) of working PicoGreen quantitation reagent were added to each well and incubated at room temperature for 2 min in the dark. Following incubation, the fluorescence intensity was measured using a CLARIOstar plate reader (BMG Labtech, Cary, North Carolina) at 480 nm excitation and 520 nm emission.

### QPCR conditions and quantitation of the PCR product

The PCR primers were designed online using the PrimerQuest Tool (Integrated DNA Technologies, Inc., Skokie, IL) or adopted from literature. The long fragment target DNAs were amplified with PrimeSTAR GXL DNA Polymerase (Clontech Laboratories Inc., Mountain View, California) using the primer sets listed in Table 1. The PCR mixture (50 μL) contained 1 x PrimeSTAR GXL PCR Buffer, 0.2 mM of each dNTP, 0.1 μM of each forward and reverse primer (Integrated DNA Technologies, Inc., Skokie, Illinois), template DNA, and 1.25 units of PrimeSTAR GXL DNA Polymerase. Cycle parameters were: denaturation, 98°C for 10 s; annealing/extension, 68°C for 10 min for 22–28 cycles.

**Table 1:** The primer sequences used in the study.

Primer	Sequence	Target	Citation
C.neo mitoLg-F	5'-GAGACATCCTAGGTCTATCTGTTCTTACTC-3'	C. neo mitoLg	Self-designed
C. neo mitoLg-R	5'-CTCTACCACTGAGCTATACTCCCTAATC-3'	C. neo mitoLg	
C. neo nLg-F	5'-GGTCGAGTCTGTGTCCTGAGAATATAA-3'	C. neo nLg	Self-designed
C. neo nLg-R	5'-GTAGAAGACGTTTAGTGGGAGAGGATAG-3'	C. neo nLg	
C. neo mitoSt-F	5'-CCGAGTTCCTTATGCGGTATTA-3'	C. neo mitoSt	Self-designed
C. neo MitoSt-R	5'-GCCGATTGAACAAGGGTTTC-3'	C.neo mitoSt	
BY4741 mitoLg-F	5’-GTGAGGGATCAACTGAAAGAGGAAAC-3’	BY4741 mitoLg	Self-designed
BY4741 mitoLg-R	5’-CCAGCAGGTACGAATAATGAGAAGAATAC-3’	BY4741 mitoLg	
BY4741 nLg-F	5’- ATCATCCCGATTGCTGCCACTAG-3’	BY4741 nLg	[20]
BY4741 nLg-R	5’- CGCTAAAATCCCGTGTATCCCTTG-3’	BY4741 nLg	
BY4741 mitoSt-F	5′-TGGAGCAGGTATCTCAACAA-3′	BY4741 mitoSt	[21]
BY4741 MitoSt-R	5′-TGTAGCTTCTGATAAGGCGA-3′	BY4741 mitoSt	

The short fragment target DNA was amplified with TaKaRa TaqDNA Polymerase Hot Start Version (Clontech Laboratories Inc., Mountain View, California) using the primer sets listed in Table 1. The PCR mixture (50 μL) contained 1 x PCR Buffer, 0.2 mM of each dNTP, 0.2 μM of each forward and reverse primer (Integrated DNA Technologies, Inc., Skokie, IL), template DNA, and 1.25 units of PrimeSTAR GXL DNA Polymerase. Cycle parameters were: denaturation, 98°C for 10 s; annealing, 52°C for 30 s, and extension, 72°C for 1 min for 18–22 cycles.

The long mitochondrial DNA and the long nuclear DNA products were characterized using BamHI, EcoRI or EcoRV restriction enzyme digestions respectively (New England Biolabs, Inc., Ipswich, Massachusetts). Full length and digested PCR products were electrophoresed on an agarose gel containing 1 x SYBR Safe DNA Stain (Thermo Fisher Scientific, Grand Island, New York) in 1 x TAE buffer. PCR yields were quantitated using the PicoGreen method as described above. This fluorescence-based method is a commercially available method to measure the DNA content with much higher sensitivity than UV-based method or gel quantification.

### Calculation of lesion frequency in long fragment DNA

DNA lesion calculation was basically carried out as described by Furda et al [10] and Seeno et al [11]. Relative amplification was calculated by dividing the amount of amplification from the radiation damaged samples (A_D_) by the amount of amplification from the un-irradiated control samples (A_C_). The lesion frequency per fragment at a particular radiation dose or a time point was calculated on the basis of a Poisson distribution (lesions/amplified fragment = -ln (A_D_/A_C_)).

### Statistical analysis

Linear regression was performed to determine the correlation of amounts of input DNA to the LR-QPCR yields. One-way ANOVA with Dunnett’s multiple comparisons tests was performed to compare the levels of short mitochondrial DNA fragment PCR yield after different radiation doses compared to the 0 Gy control. Student’s t test was performed to compare the DNA lesions in H99S vs H99 at the same time points after radiation exposure. Statistical analysis was performed using GraphPad Prism 7.03 software. All values of p < 0.05 were considered as significant differences.

## Results

### Primer design for detection of DNA damage in *C*. *neoformans and S*. *cerevisiae*

*C*. *neoformans* H99 mitochondrial DNA (mtDNA) consists of a 24.9-kb long circular molecule (Genbank accession number NC_018792), and the A+T content of this genome is 65%. PCR primers were designed to amplify a long target (mitoLg, 15.3 kb) and a short target (mitoSt, 207 bp) in mtDNA; primers were also designed to amplify a long nuclear DNA target (nLg, 17.6 kb) on chromosome 1. PCR primers for *S*. *cerevisiae* BY4741 long nuclear DNA target (nLg, 16.4 kb) and short mitochondrial DNA target (mitoSt, 154 bp) amplification were adopted from published methods [20, 21]. The PCR primers to amplify the *S*. *cerevisiae* BY4741 long mtDNA target (mitoLg, 14.6 kb) were designed according to the BY4741 mitochondrial DNA sequences (Genbank accesion number JRIS01000397.1). The primer sequences were listed in Table 1.

### Quantitative amplification of mitochondrial and nuclear DNA fragments

**Fig 1:**
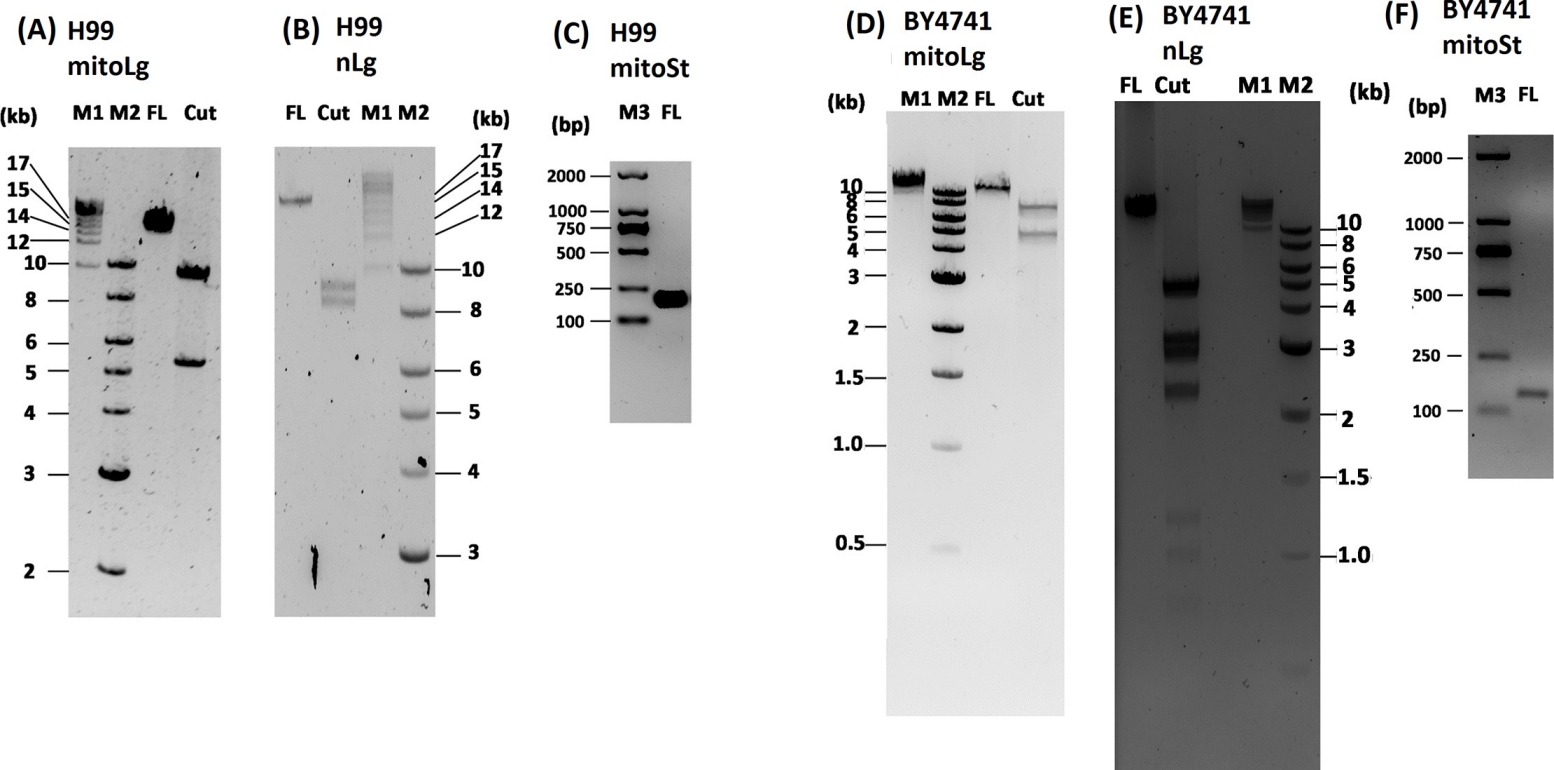
Amplification of long mtDNA and nDNA and short mtDNA from *C*. *neoformans* H99 and *S*. *cerevisiae* BY4741 DNA extract. (A and D) mitoLg, long mitochondrial DNA fragments from H99 and BY4741, (B and E) nLg, long nuclear DNA fragments from H99 and BY4741, (C and F) mitoSt, short mitochondrial DNA fragments from H99 and BY4741. M1, M2 and M3 are different size DNA ladders. FL, full length; Cut, restriction enzyme digested full length DNA.

The long range PCR was performed using the TaKaRa PrimeSTAR GXL DNA polymerase since it was shown to amplify almost all amplicons with different sizes and Tm values under identical PCR conditions [13]. In *C*. *neoformans* H99, after electrophoresis the long mitochondrial PCR fragment was a single band of 15 kb as expected; furthermore, restriction enzyme (BamHI) digestion of the long mitochondrial PCR fragment generated two bands of 9.7 and 5.5 kb as expected (Fig 1A). The long nuclear PCR fragment was a single band of 17 kb as expected; restriction enzyme (EcoRI) digestion of the long nuclear PCR fragment generated two bands of 8.4 and 9.2 kb as expected (Fig 1B). The short mitochondrial PCR fragment was about 200 bp as expected (Fig 1C). In *S*. *cerevisiae* BY4741, after electrophoresis the long mitochondrial PCR fragment was a single band of 14 kb and restriction enzyme (EcoRV) digestion of the long mitochondrial PCR fragment generated two bands of 5.4 and 9.1 kb as expected (Fig 1D). The long nuclear PCR fragment was a single band of 16 kb and restriction enzyme (EcoRI) digestion of the long nuclear PCR fragment generated six visible bands of 5.0, 3.2, 2.9, 2.3, 1.2, 0.9 and 0.7 kb as expected (1 small band of 0.7 kb was invisible on the gel) (Fig 1E). The short mitochondrial PCR fragment was about 150 bp as expected (Fig 1F).

**Fig 2:**
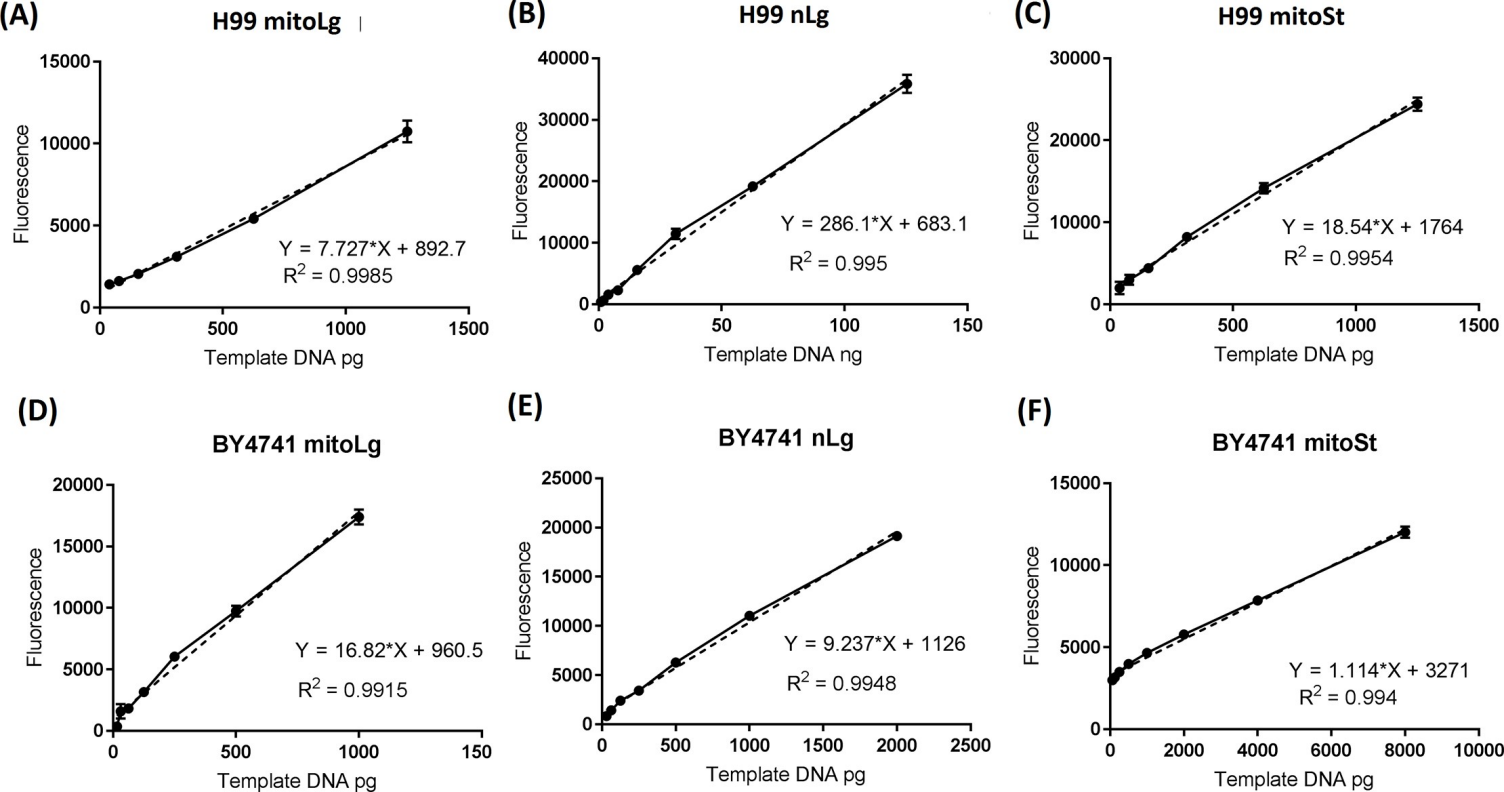
Quantitative amplification of target DNA fragments in LR-QPCR with respect to the amount of template DNA. DNA isolated from *C*. *neoformans* H99 and *S*. *cerevisiae* BY4741 were serially diluted with nuclease-free water for use in PCR. PCR of each sample was performed in triplicate for 26 cycles (long mitochondrial and nuclear DNA) and 20 cycles (short mitochondrial DNA), and the PCR products were quantified using the PicoGreen assay. (A and D) long mitochondrial DNA fragments from H99 and BY4741, (B and E) long nuclear DNA fragments from H99 and BY4741, (C and F) short mitochondrial DNA fragments from H99 and BY4741. The solid lines connect all of data points. The dotted lines are the linear regression lines. Results were from three independent experiments with triplicate samples in each experiment (3 samples/point/experiment x 3). Mean ± S.E.M.

Experiments were carried out to determine the amounts of template DNA and PCR cycle numbers for quantitative PCR, to make sure that the PCR amplification yields are directly proportional to the starting amount of DNA template. The target DNAs (mitoLg, nLg and mitoSt) were amplified from serially diluted template DNA with different PCR cycle numbers. PCR products were quantified using PicoGreen assay. The *C*. *neoformans* mitoLg and mitoSt PCR product yield was proportional to the amount of template DNA in the range from 39 pg to 1250 pg DNA, with 26 cycles and 20 cycles (R^2^ = 0.9985 and R^2^ = 0.9954), respectively. (Fig 2A & 2C). The *S*. *cerevisiae* BY4741 PCR product yield was proportional to the template DNA in the range from 16 pg to 1000 pg (mitoLg) and 62 pg to 8000 pg (mitoSt), with 26 cycles and 20 cycles (R^2^ = 0.9915 and R^2^ = 0.9940), respectively. (Fig 2D & 2F). The nLg PCR product yield was proportional to the template DNA amount of *C*. *neoformans* in the range from 1 ng to 125 ng with 26 cycles (R^2^ = 0.9950) (Fig 2B) and 32–2000 pg template DNA from BY4741 with 26 cycles (R^2^ = 0.9948) (Fig 2E).

### Quantitative detection of DNA damage in *C*. *neoformans* H99 *and S*. *cerevisiae* BY4741 exposed to γ-radiation

**Fig 3:**
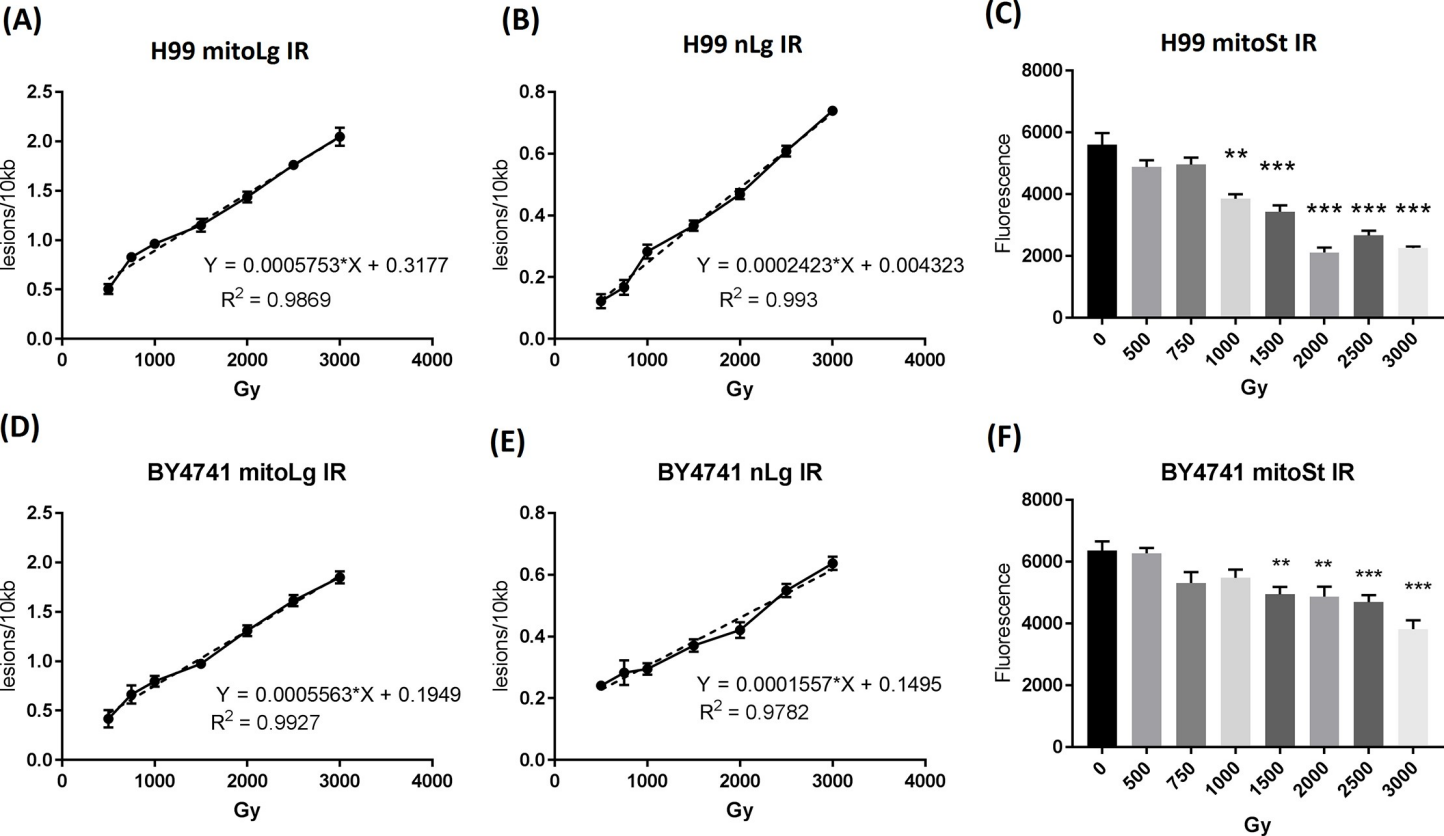
Quantitative detection of DNA damage in *C*. *neoformans* H99 and *S*. *cerevisiae* BY4741 exposed to ionizing radiation using the long mitochondrial DNA and nuclear DNA fragments. H99 and BY4741 cells were exposed to ionizing radiation from doses of 500 Gy to 3000 Gy. Irradiated cells were immediately frozen at -80^°^C and DNA extraction was performed as described in the Material and Methods. PCR products were quantified using the PicoGreen assay. DNA lesions were calculated according to the formula (lesions/amplified fragment = -ln (A_D_/A_C_)). The solid lines are lines connecting each data point. The dotted lines are the linear regression lines. (A and D) long mitochondrial DNA fragment lesions, (B and E) long nuclear DNA fragment lesions, (C and F) short mitochondrial DNA fragment PCR yield. Results were from three independent experiments with triplicate samples in each experiment (3 samples/point/experiment x 3). Mean ± S.E.M. **, p<0.01; ***, p<0.001.

*C*. *neoformans* H99 and *S*. *cerevisiae* BY4741 cells were exposed to different doses of γ-radiation from 500 Gy to 3,000 Gy and cells were frozen at -80^°^C right after radiation. DNA was extracted as previously described. LR-QPCR was performed to measure the DNA lesions in the irradiated cells using the established conditions (1 ng template DNA was used for the H99 mitoLg and mitoSt LQ-PCR assay, and 30 ng template DNA was used for the H99 nLg LR-QPCR assay. For BY4741, 1 ng template DNA was used for the mitoLg and nLg LR-QPCR assay, and 4 ng template DNA was used for the mitoSt LR-QPCR assay). At the studied radiation doses, both the mitochondrial and nuclear DNA lesions in the irradiated H99 and BY4741 cells had a linear relationship with the radiation doses (H99: R^2^ = 0.9869 and R^2^ = 0.9930; BY4741: R^2^ = 0.9927 and R^2^ = 0.9782; respectively) as shown in Fig 3. There were more radiation-induced DNA lesions in the mtDNA than the nuclear DNA at the same radiation doses.

The short mtDNA fragments were also amplified to monitor the mitochondrial numbers in H99 and BY4741. Because they are only 207 and 154 bases long, the mitoSt fragments are less likely to capture radiation-induced DNA damage, therefore the mitoSt can reflect the mitochondrial number. As shown in Fig 3C and 3F, the mitoSt amplification was lower than the 0 Gy group starting from 500–750 Gy and statistically lower starting from 1000 Gy in H99 and 1500 Gy in BY4741, suggesting that radiation doses above these levels significantly decrease the number of mitochondria. Because of this, the long mtDNA fragment values were not adjusted using the short mtDNA fragment values.

### Elevated DNA damage may explain the radiation sensitivity of *C*. *neoformans* H99 compared to *C*. *neoformans* H99S

**Fig 4:**
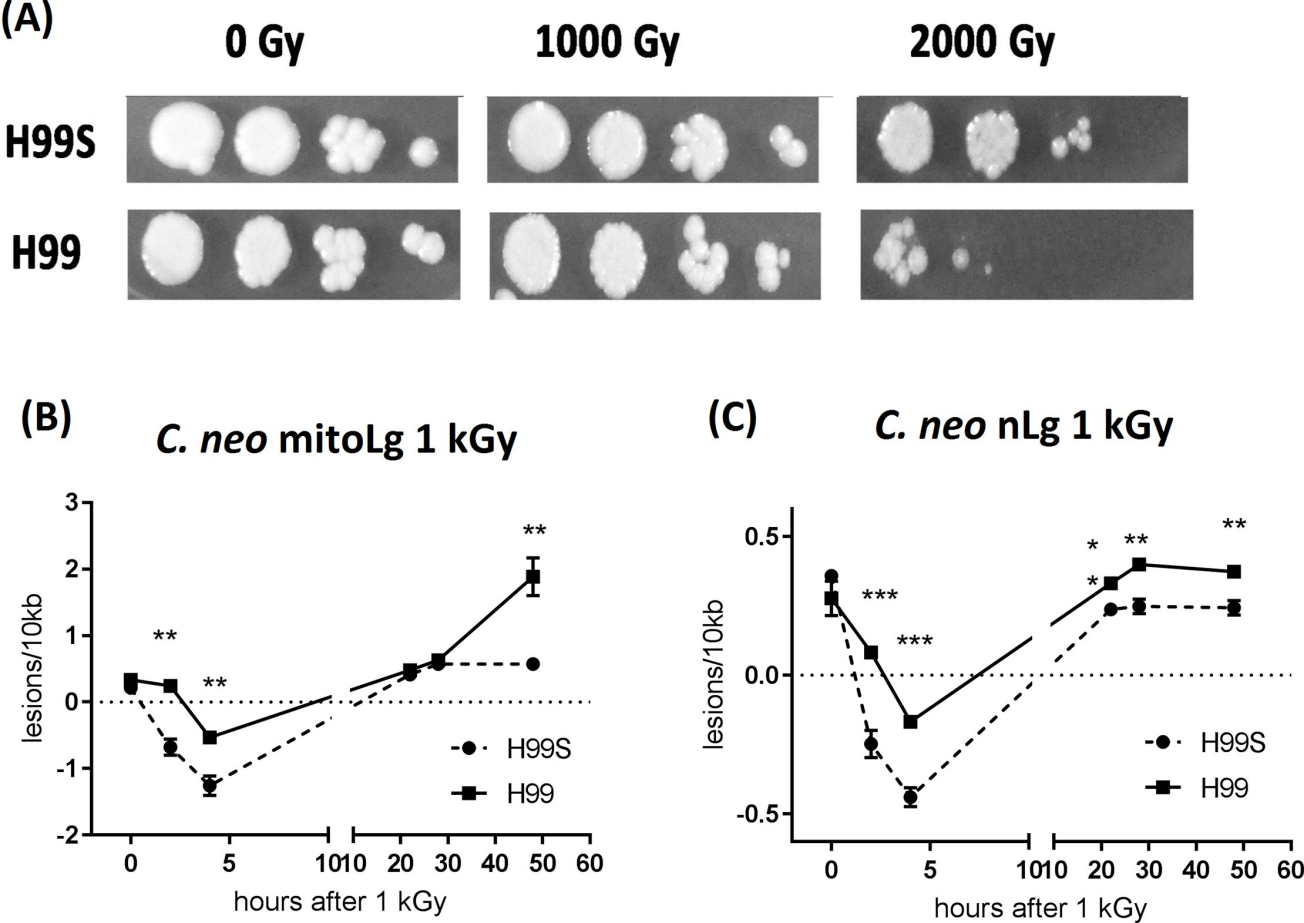
Different radio-sensitivity of *C*. *neoformans* H99S vs H99 strains and their DNA damage progression after 1 kGy exposure. (A) H99S and H99 cells were cultured in YPD overnight, and then 10-fold serially diluted (10^2^ to 10^5^) in PBS and 3 μL of diluted solution was spotted onto the YPD plates. Cells were exposed to the indicated doses of γ-radiation and then further incubated at 30^°^C for 5 days. Image was from one representative experiment of three independent experiments. (B) & (C) DNA damage progression of *C*. *neoformans* H99S and H99 cells after 1 kGy γ-radiation measured by mitochondrial DNA and nuclear DNA LR-QPCR, respectively. After radiation, cells were harvested at selected time points and DNA was extracted and PCR was performed using 1 ng DNA and 30 ng DNA for the long mitochondrial DNA fragment and long nuclear DNA fragment respectively. Results were from three independent experiments with triplicate samples in each experiment (3 samples/point/experiment x 3). Mean ± S.E.M. **, p<0.01; ***, p<0.001.

H99S strain has been reported to have different phenotypic responses to environmental stresses and anti-fungal drugs compared to H99 strain [16]. To study whether they also have different radiation sensitivity, H99S and H99 fungal cells were exposed to 1000 and 2000 Gy γ- radiation (Fig 4A). H99S and H99 cells grew similarly after 1000 Gy. However, H99 cells exhibited severe growth reduction compared to H99S after 2000 Gy. To test whether their DNA damage levels are different after radiation exposure, we measured the DNA damage using the developed LR-QPCR method. Results are showed in Fig 4B & 4C. The radiation-induced mitochondrial and nuclear DNA damage were observed in both H99S and H99 cells at similar levels right after 1000 Gy radiation, whereas significant differences of these DNA damage levels were observed in H99S vs H99 cells 2 and 4 hours after 1000 Gy radiation. Interestingly, H99S at 2 and 4 hours and H99 at 4 hours had more mitochondrial and nuclear DNA PCR amplification (as shown by the less DNA lesions) compared to their respective un-irradiated controls. At 22 and 28 hours after radiation exposure, levels of the mtDNA damage were similar in H99S vs H99. However, 48 hours after radiation exposure the mtDNA damage level in the H99 strain was significantly increased in comparison with the H99S strain. Furthermore, at 22, 28 and 48 hours after radiation exposure, the nuclear DNA damage levels in the H99 were all significantly higher than in the H99S strain.

## Discussion

The goal of the current study is to develop a simple and sensitive method to measure radiation-induced DNA damage (Figs 1 & 2). Compared to the currently used methods to measure radiation-induced DNA damage, LR-QPCR method doesn’t need special equipment, and only needs about 5 hour to run the assay. Another advantage is that the LR-QPCR is very sensitive, only needs 1 ng DNA to measure mitochondrial DNA damage and 30 ng DNA to measure the nuclear DNA damage in the *C*. *neoformans* and 1 ng DNA for both the mitochondrial and nuclear DNA damage in BY4741. DNA can be extracted from exposed samples and stored at -20^°^C for later analysis. The LR-QPCR method to measure DNA damage is likely universal and not restricted to one genetic background as shown it works in two genetically different species (*C*. *neoformans* and *S*. *cerevisiae*). Unlike the PFGE and comet assay, the LR-QPCR method can be performed in a high-throughput manner, which may benefit the screening of radiation countermeasures that protect cells from ionizing radiation.

Using this method, we showed that the radiation-induced DNA damage in *C*. *neoformans* and *S*. *cerevisiae* is linearly correlated to the exposed radiation doses they exposed (Fig 3). This is in agreement with literatures showing that radiation induced quantitative DNA damage [3, 4]. Unlike previous methods that are difficult to quantify the frequency of DNA damages, the LR-QPCR method can easily quantify the DNA lesions induced by radiation. Therefore, the method may have the potential to be used as a radiation biodosimetry to predict radiation doses in animals or humans.

Our data showed that there were more radiation-induced DNA damages in mitochondrial DNA than in nuclear DNA (Figs 2–4) at the same radiation doses or time points; this is consistent with the previous reports that mitochondrial DNA damage is more extensive and persistent than nuclear DNA damage following oxidative stresses both in cells [22, 23] and in tissues [24]. There are more damages in mtDNA than nDNA because of multiple reasons: mtDNA is close to the respiratory chain, which produces a lot of oxidants; mtDNA doesn’t have histone-like protective proteins, and there are limited DNA repair machineries for mtDNA compared to nDNA [25, 26]. Since there are hundreds of mitochondria in each cell and mtDNA has more damage than nDNA, the LR-QPCR for determining the mtDNA damage may be used as a sensitive and specific method for radiation damage.

There was more DNA amplification in *C*. *neoformans* at 4 hour after radiation exposure as shown by lesion rate decrease in Fig 4, which may suggest that there were some chromosome remodeling happened at this time point. This observation is consistent with the “access-repair-restore” model explaining chromatin remodeling after DNA damage [27, 28]. In this model, chromatin organization is modified (histone modifications may be one of the primary mechanisms) after DNA damage, thus allowing the access of repair machineries to DNA lesions. It is possible that the chromatin modification may allow better access for the PCR polymerase in the PCR reaction, thus explaining the more PCR amplification at this time point after radiation compared to the un-irradiated control. Furthermore, one interesting finding from our data in Figs 3 and 4 is that the DNA damage measured by LR-QPCR right after radiation injury are similar in *C*. *neoformans* H99, *S*. *cerevisiae* BY4741 and *C*. *neoformans* H99S even though these three strains have different radiation sensitivity, implying that radiation causes similar levels of DNA damage right after radiation exposure even in different species. What matters most may be the different repairing systems in these organisms. The data is in agreement with the previous report showing that vastly different organisms have similar levels of DNA damage after radiation even though they have very different radiation sensitivities [4].

To summarize, we have developed a simple, sensitive LQ-QPCR method to measure radiation-induced DNA damage in *C*. *neoformans and S*. *cerevisiae*. This method can quantitatively measure both mitochondrial and nuclear DNA damages and monitor their progresses.
